# TREM-1 Modulates Dendritic Cells Maturation and Dendritic Cell-Mediated T-Cell Activation Induced by ox-LDL

**DOI:** 10.1155/2022/3951686

**Published:** 2022-05-21

**Authors:** Yun Kai Wang, Jie Wang, Feng Hua, Yun Li Shen, Lu Han, Jie Yun You, Wei Wei, Chun Yu Zhang, Xiang Dong Liu, Qi Zhang

**Affiliations:** ^1^Department of Cardiology, Shanghai East Hospital, Tongji University School of Medicine, Shanghai, China; ^2^Department of Cardiology, Taihe Hospital, Hubei University of Medicine, Shiyan, Hubei, China; ^3^Department of Respiratory Medicine, Affiliated Huzhou Hospital, Zhejiang University school of Medicine, Huzhou, Zhejiang Province, China; ^4^Qingdao University Medical College, Qingdao, China; ^5^Department of Cardiology, Zhongshan Hospital, Fudan University, Shanghai, China; ^6^Department of Emergency, Shanghai East Hospital, Tongji University School of Medicine, Shanghai, China

## Abstract

Atherosclerosis is a chronic inflammatory disease. The triggering receptor expressed on myeloid cells-1 (TREM-1) plays a crucial role in inflammatory diseases; recently, it was identified as a major upstream proatherogenic receptor, but its mechanism is still unclear. In this study, we explore the role of TREM-1 on dendritic cells maturation and inflammatory responses induced by ox-LDL and its possible mechanism. Human dendritic cells were differentiated from blood monocytes and treated with ox-LDL. Naive autologous T cells were cocultured with pretreated DCs or treated directly. The expression of TREM-1 and inflammatory factors were evaluated by real-time PCR, western blot, and ELISA methods. And the expression of immune factors to evaluate the DCs maturation and T-cell activation were determined by the FACS. Our study showed that ox-LDL induced TREM-1 expression, DC maturation, and T-cell activation. T cells exposed to ox-LDL-treated DCs produced interferon-*γ* and interleukin-17 (IL-17). Blocking TREM-1 suppressed the DC maturation, showing lower expression of CD1a, CD40, CD86, CD83, and HLA-DR, and limited their production of tumor necrosis factor-alpha (TNF-*α*), IL-1*β*, IL-6, and monocyte chemoattractant protein-1 (MCP-1), meanwhile increased transforming growth factor-*β*(TGF-*β*) and IL-10 production. Ox-LDL induced miR-155, miR-27, Let-7c, and miR-185 expression; however, TREM-1 inhibiting decreased miRNA-155 expression. Furthermore, silencing miRNA-155 restores SOCS1 repression induced by ox-LDL. Experiments with T cells derived from carotid atherosclerotic plaques or healthy individuals showed similar results. Our results uncover a new link between ox-LDL and TREM-1 and may provide insight into this interaction in the context of atherosclerosis.

## 1. Introduction

It is now well established that atherosclerosis is a chronic inflammatory disease of the vessel wall. Dendritic cells (DCs) serve as a key player in the pathogenesis of atherosclerosis via antigen presenting, lipid internalization, inflammatory cytokine secretion, and inflammation response induction [1–3]. Previous studies documented that DCs had compact adhesion to the endothelium in atherosclerotic lesions, leading to plaque rupture, plaque hemorrhage, and thrombosis, finally causing acute cardiovascular events following oxidized low-density lipoprotein (ox-LDL) [4–6]. Although there is considerable evidence that DCs control lipid uptake and cholesterol metabolism and modulate adaptive immune responses in atherosclerosis, a clear attribution to bona fide DCs and the exact molecular mechanisms engaged are mostly still unclear [7].

Triggering receptor expressed on myeloid cells-1(TREM-1), an immunoglobulin cell surface receptors, acting as an amplifier of inflammation, is involved in the control of acute and chronic inflammatory responses [8–13]. TREM-1 is expressed on myeloid cells and is verified to play an important role in the modulation of host immune responses by promoting mononuclear cell differentiation to DCs and accelerating antigen presentation [14, 15]. Clinical studies have detected sTREM-1, a soluble form of TREM-1, in the serum of patients with sepsis, coronary artery disease, diabetes, and obesity [16–18]. Blocking TREM-1 has been suggested to suppress inflammatory cytokine production and alleviate the progress of infectious and noninfectious diseases [19–21], including atherosclerosis [22]. However, to date, the precise role of TREM-1 and its downstream factors orchestrating atherosclerosis inflammation has not been clarified.

Multiple reviews have highlighted miR dysregulation in atherosclerosis progression [23–25]. MiR-155, a typical multifunctional miRNA, is emerging as a novel regulator involved in the inflammation signaling pathway in the pathogenesis of atherosclerosis [27–29]. Although the functional relevance of DC miR-155 expression is unclear, studies have indicated that miR-155 shows both anti- and proinflammatory effects by regulating TAB2 and SOCS-1, respectively [30].

Herein, in this study, we examined whether DCs TREM-1 regulates DC maturation, T-cell activation, and inflammatory responses and whether this involvement is dependent on miR-155 and are mediated by downregulation of suppressor of cytokine signaling-1 (SOCS-1) a key miR-155 target.

## 2. Materials and Methods

### 2.1. Reagents

Lipofectamine® 2000 Transfection Reagent and TREM-1 siRNA were purchased from Invitrogen (Carlsbad, USA). TRIzol, LPS, and human LDL were purchased from Sigma-Aldrich (St. Louis, USA). LP17 (LQVTDSGLYRCVIYHPP) and a sequence-scrambled control-peptide (TDSRCVIGLYHPPLQVY) were produced by GL Biochem (Shanghai) LTD, as described by Gibot et al. [18]. The peptides were obtained with good yields (>99%), purified, and confirmed by preparative chromatography. These peptides were free of endotoxin. The anti-CD14 and CD4 magnetic beads were purchased from Miltenyi Biotec (Bergisch Gladbach, Germany). The ELISA kits, CD1a-FITC, CD40-FITC, CD86-FITC, CD83-FITC, CD14-FITC, and HLA-DR-PE antibodies were purchased from BD (Franklin Lakes, USA). The anti-TREM-1 primary antibody and Human sTREM-1 ELISA kit were purchased from R&D (HK, China). The anti-SOCS1and GAPDH primary antibodies were purchased from Cell Signaling (Denver, USA). The cDNA Synthesis Kit and Premix Ex Taq SYBR Green PCR Kit were purchased from Takara (Shiga, Japan). Recombinant human granulocyte macrophage colony-stimulating factor (GM-CSF) and interleukin 4 (IL-4) were purchased from R&D Systems (Minneapolis, USA).

### 2.2. Preparation of ox-LDL

Human LDL was dialyzed against PBS (50 mM phosphate buffer, pH 7.4, 0.15 M NaCl, 0.01% NaN3, and 6.67 *μ*M CuSO4) at 37°C for 24 h. After the incubation was terminated by EDTA (0.5 mg/mL), the preparations were dialyzed and preserved in nitrogen-filled tubes. The extent of LDL oxidation was determined using the thiobarbituric acid-reactive substance (TBARS) test [31].

### 2.3. Generation of Human moDCs

This study was approved by the Institutional Review Board of Shanghai East Hospital, Tongji University (Shanghai, China). Healthy donors and patients provided gave their written consent for the use of blood donation for research purposes.

As previously described [32], human PBMCs were purified from whole blood directly obtained from healthy donors and patient using a Ficoll-Paque PLUS density gradient centrifugation (2,400 rpm, 20 min). Next, CD14^+^ monocytes isolated from the PBMC fraction with MidiMACS Separator separation columns and anti-CD14 Abs coated on magnetic beads (Miltenyi Biotec, Bergisch Gladbach, Germany). CD14^+^ monocytes were cultured at 1 × 106 cells/mL in the presence of GM-CSF (100 ng/mL) and IL-4 (50 ng/mL) (Miltenyi Biotec) in RPMI 1640 25 mM HEPES GlutaMAX supplemented with 10% FCS, antibiotics, and 1% pyruvate at 37°C in humidified air containing 5% CO_2_. The medium was replaced every 2 days. Within 6 d, monocytes differentiated into moDCs with an immature phenotype [32].

### 2.4. Treatment of moDCs

On day 6, immature moDCs were stimulated by 25, 50, or 100 *μ*g/mL of native or oxidative LDL (100 ng/mL LPS treatment as a positive control) for 24 h, depending on experiments. In some experiments, moDCs were pretreated for 24 h with either control RNA or siRNA targeted human TREM-1, miR155 mimics, miR155 inhibitors and controls.

### 2.5. Allogeneic Cocultures of moDCs and T Cells

CD4^+^ T cells were isolated from PBMCs by positive selection with MidiMACS separation columns and anti-CD4 Abs coated on magnetic beads (Miltenyi Biotec). These T cells were confirmed to have purity >95%, based on CD4 expression evaluated by flow cytometry (561841, RPA-T4; BD Biosciences). CD4+ T lymphocytes were labeled with 0.5 mM CFSE (Invitrogen), following the manufacturer's instructions. MoDCs were stimulated with native or oxidative LDL for 24 h and then washed and cocultured with allogeneic CD4^+^ T cells at a 1 : 10 DC/T cell ratio for 5 d, in RPMI 1640 GlutaMAX supplemented with 10% fetal bovine serum (Gibco, US origin) in round-bottom 96-well plates. CFSE fluorescence was analyzed using flow cytometry with an Attune NxT (Invitrogen) using FlowJo software (version 10; FlowJo). Proliferating T cells corresponded to low CFSE fluorescence [32, 33].

### 2.6. T-Cell Differentiation and Suppression

CD4^+^ T cells were generated as above. With anti-CD45RO-coupled magnetic beads and LD-negative selection columns (Miltenyi Biotech), we depleted CD45RO^+^ cells from CD4+ T cells. In these purified cells, the percentage of CD4^+^CD45RO-CD45RA^+^T cells was >90%. T cells stimulated with ox-LDL-treated DCs were referred to as T [DC (ox-LDL)], and T cells stimulated with (siR-TREM-1+ ox-LDL)- and (LP17 + ox-LDL)-treated DCs were referred to as T[DC (siR-TREM-1 + ox-LDL)] and T[DC (LP17 + ox-LDL)].

In 1 mL of X-vivo 15 medium, 1 × 10^5^ autologous DCs were cocultured with 1 × 10^6^ CD4 + CD45RO-T cells. After 6 days, 20 U/mL recombinant human IL-2 was present, and then cells were cultured for another 7 days. Thirteen days after initiation of the culture, T cells were collected, washed, and assessed for their function.

To determine the suppressive function of these T cells, autologous CD4^+^CD45RO^−^ T cells prelabeled with CFSE (5 *μ*mol/L) were cultured with mature DCs (10 : 1; 10^5^T:10^4^DC), in the absence or presence of T (DC), T (DC [ox-LDL]), T (DC [siTREM-1 + ox-LDL]) cells, or (DC [LP17 + ox-LDL]) (1 : 1 ratio). At 5 days, cells were collected, and CFSE expression was tested by flow cytometry. The results are expressed as the percentage of proliferating CFSE low CD4+ cells [32–34].

### 2.7. Plaque T-Cell Isolation

This study was preapproved by the research ethics committee of Shanghai East Hospital, Tongji University (Shanghai, China), and in accordance with the Declaration of Helsinki. All subjects gave their written informed consent before entering the study.

Through carotid endarterectomies, we obtained carotid endarterectomy specimens with advanced type IV atherosclerotic lesions, with the help of surgeons from Shanghai East Hospital, Tongji University, China. These patients reported on recent clinical events of minor stroke or transitory ischemic attacks.

As described in a previous report [32], carotid plaques were finely chopped into small pieces and were digested with collagenase D in RPMI-1640 containing DNase at 37°C for 4 hours. The dissociated plaque cells were filtered with a 100-*μ*m cell strainer to remove fat and tissue debris. Next T cells were purified with the EasySep T-cell enrichment kit (STEMCELL Technologies). DCs obtained from the peripheral blood of the plaque donors were treated with or without ox-LDL as described above and thereafter cocultured with plaque T cells.

### 2.8. Real-Time Reverse Transcription PCR (RT-PCR)

The expression of TREM-1, TXB2, GATA3, RORC, Foxp3, and SOCS1 were detected by SYBR Green-based real-time RT-PCR. Using TRIzol and cDNA Synthesis Kit, total RNA was extracted and converted to cDNA according to the manufacturer's instructions. RT-PCR was performed using an ABI 7500 system with Premix Ex Taq SYBR Green chemistry.

### 2.9. Western Blot

After lysis on ice for 45 min, the samples were quantified using Bradford. Then the samples with equal concentrations of protein were subjected to SDS-PAGE, transferred to NC membranes, and blocked using 5% nonfat milk. At 4°C, the cells were incubated with primary antibodies overnight and subsequently coated with HRP-conjugated secondary antibodies for 1 h at room temperature. The immunoblot was visualized using enhanced chemiluminescence.

### 2.10. Flow Cytometry

After 24 h of treatment with native or oxidative LDL, moDC viability determined by MTT and phenotype were analyzed by flow cytometry.

### 2.11. siRNA Transfection and Plasmid Transfection

According to the manufacturer's protocol, cells were transfected with either control RNA or siRNA targeted human TREM-1, miR155 mimics, miR155 inhibitors, and controls (RiboBio, China) with the Lipofectamine® 20009((Life Technologies, Inc., Carlsbad, CA) on day 5 for 24 hours and then phosphate buffer solution (PBS), ox-LDL (50 *μ*g/mL) were added to the medium for another 24 hours. The transfection rate was routinely ≥80%, and the viability of the DCs was not influenced by this method (data not shown).

### 2.12. TREM-1 Blocking

To investigate the role of TREM-1, cells were treated with LP-17 (100 ng/mL) for 24 h as well as isotype matched controls in the presence of ox-LDL (50 *μ*g/mL).

### 2.13. Enzyme-Linked Immunosorbent Assay

After a 24-hour treatment as indicated, the supernatants from DCs were collected, and the amounts of TNF-*α*, IL-10, IL-12, IL-1*β*, TGF-*β*, IL-6, CCL5, and CCL17 were determined according to the manufacturer's instructions (R&D Systems).

### 2.14. Statistical Analysis

All data were expressed as mean ± SD, and the differences were performed using SPSS 20.0. One-way ANOVA followed by Tukey's HSD test was used to compare the significant differences. Values of *p* < 0.05 were considered statistically significant.

## 3. Results

### 3.1. ox-LDL-Induced DCs TREM-1 Expression

**Figure 1:**
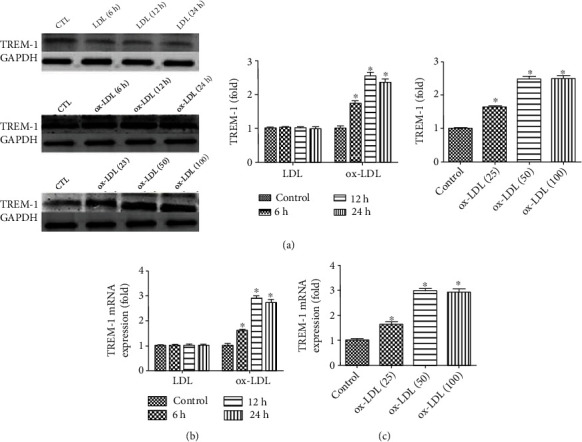
ox-LDL-induced DCs TREM-1 expression. (a) DCs at day 6 were treated with LDL or ox-LDL at different time (0, 6, 12, and 24 h) or at different concentration (25, 50, or 100 *μ*g mL^−1^) for 24 h. Then, the protein levels of TREM-1 were determined. (b) DCs at day 6 were treated with LDL or ox-LDL at 50 *μ*g mL^−1^ at 0, 6, 12, and 24 h, and then the mRNA levels of TREM-1 were determined. (c) DCs at day 6 were treated with ox-LDL at 25, 50, or 100 *μ*g mL^−1^ for 24 h, and then the mRNA levels of TREM-1 were determined. ∗*p* < 0.05, compared with control cells; ^#^*p* < 0.05, compared with ox-LDL-treated cells.

To investigate the effects of ox-LDL on TRME-1 expression, imDCs were exposed to ox-LDL (50 *μ*g/mL) for 0, 6, 12, and 24 h, and then TREM-1 protein and RNA levels were evaluated by western blotting and real-time PCR. As shown in Figure 1, incubation with ox-LDL induces a marked increase in TREM-1 mRNA and protein levels, whereas native LDL did not (Figures 1(a)–1(c)).

**Figure 2:**
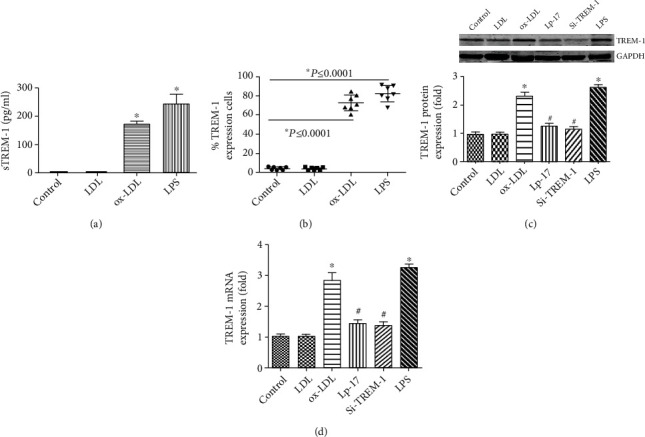
ox-LDL-induced DCs TREM-1 expression with or without LP17 and SiTREM-1. At day 6, DCs were treated with LDL or ox-LDL for 24h; 100 ng/mL LPS treatment is a positive control. (a) Cell-free supernatants were harvested and assayed for sTREM-1 content by ELISA (b) TREM-1 surface expression. DCs were stained with anti-CD1a-allophycocyanin and anti-TREM-1-PE Abs and analyzed by flow cytometry. Cells were electronically gated according to their light scatter properties to exclude cell debris. Data are expressed as a percentage of TREM-1^+^cells within CD1a^+^DCs and ox-LDL-DCs generated from healthy volunteer donors. (c, d) For downregulation of TREM-1, DCs was transfected with TREM-1 silencer (SiTREM-1) or treated with LP17 at day 6, and ox-LDL was added 24 h after transfection and further incubated for 24 h. Then, the mRNA levels and protein levels of TREM-1 were determined. ∗*p* < 0.05, compared with control cells; ^#^*p* < 0.05, compared with ox-LDL treated cells.

TREM-1 surface expression was measured by flow cytometry, and the soluble form of TREM-1 (sTREM-1) was determined by ELISA. Incubation with ox-LDL induce a marked elevation in the number of TREM-1^+^ DCs and sTREM-1 levels; in contrast, native LDL did not (Figures 2(a)–2(d)).

### 3.2. TREM-1 Mediated DC Maturation and Inflammation

**Figure 3:**
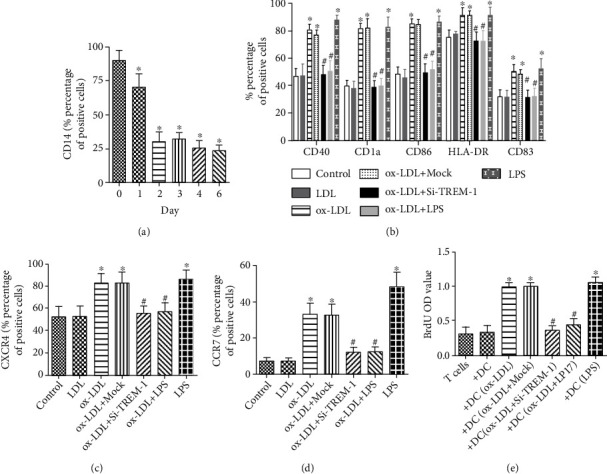
TREM-1 plays an important role in DCs maturation and subsequent T-cell proliferation induced by ox-LDL. (a) The process of monocytes differentiated into immature DCs (imDC), at 0, 1, 2, 3, 4, 5, and 6 days; the expressions of CD14 were determined by flow cytometry. (b) At day 6, immature DCs were treated with LDL or ox-LDL for 24 h, and the expressions of CD1a, CD40, CD86, CD83, and HLA-DR on DCs were analyzed by flow cytometry. 100 ng/mL LPS treatment is a positive control. (c, d) CXCR4 and CCCR7 on DCs were analyzed by flow cytometry. 100 ng/mL LPS treatment is a positive control. (e) T- cell proliferation determined by BrdU incorporation assay. After the indicated treatment, DCs at day 7 were washed and cocultured in triplicate for 5 days with autologous T cells. 10 *μ*mol L−1 BrdU was present in the last 16 h. Proliferative response was evaluated as OD value. Results represent the mean ± SD of seven-eight experiments. ∗*p* < 0.05, compared with control cells; ^#^*p* < 0.05, compared with ox-LDL treated cells.

Treatment with ox-LDL markedly increased the expression of CD1a, CD40, CD86, CD83, and HLA-DR in immature DCs. LPS treatment shows similar results (Figures 3(a)–3(b)). These expression of typical surface molecules indicated that DCs grow from immature to mature.

**Figure 4:**
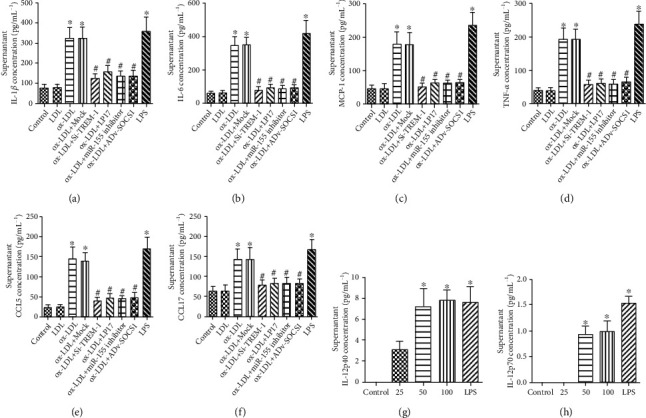
TREM-1 regulated ox-LDL-induced DCs inflammation. (a–f) At day 6, DCs were transfected with or without TREM-1 silencer (SiTREM-1) or relevant scrambled siRNA or treated with LP17, and ox-LDL was added 24 h after transfection and further incubated for 24 h. DC supernatants were collected, and cytokine production were tested by ELISA. (g, h) At day 6, DCs were treated with ox-LDL at different concentrations (25, 50, or 100 *μ*g mL^−^^1^) for 24 h, IL-12 was determined by ELISA, and 100 ng/mL LPS treatment is a positive control. Results represent the mean ± SD of seven-eight experiments. ∗*p* < 0.05, compared with control cells; ^#^*p* < 0.05, compared with ox-LDL-treated cells.

As the previous study, we also found that ox-LDL induced high expression of TNF-*α*, IL-1*β*, IL-6, IL-12, and MCP-1 (Figure 4), compared to the control group.

To explore the role of TREM-1 in ox-LDL-induced DC maturation and inflammation, we analyzed the changes in CD1a, CD40, CD83, CD86, and HLA-DR, and cytokine production in DCs by inhibiting TREM-1. As shown in Figures 3(a)–3(d), treatment with SiTREM-1 or LP-17 significantly repressed ox-LDL-induced upregulation of typical surface molecules on DCs, meanwhile, inhibited the production of TNF-*α*, IL-1*β*, IL-6, and MCP-1 (Figure 4).

### 3.3. T-Cell Activation Induced by ox-LDL-Treated DCs Is Alleviated by TREM-1 Inhibition

Mature DCs are considered to be the major stimulation to naïve T cells. Here, we explored the role of TREM-1 in T-cell proliferation. DCs were treated with LP17 or TREM-1 siRNA transfection and/or ox-LDL and applied in DC-T-cell coculture experiments. We found that ox-LDL-treated DCs stimulated T-cell proliferation (Figure 3(g)). On the contrast, pretreatment with SiTREM-1 or LP-17, T-cell proliferation stimulated by ox-LDL-treated DCs was markedly suppressed (Figure 3(e)).

### 3.4. Blocking TREM-1 Induces Th1 Responses and Increased Th2 Responses

Mature DCs acquire the ability to differentiate naive CD4^+^ T cells into Th1, Th2, or Th17 cells or Treg cells. To ascertain the mechanism by which TREM-1 modulates the immune response, selected Th1/Th2 cytokines were analyzed by ELISA or real-time PCR.

**Figure 5:**
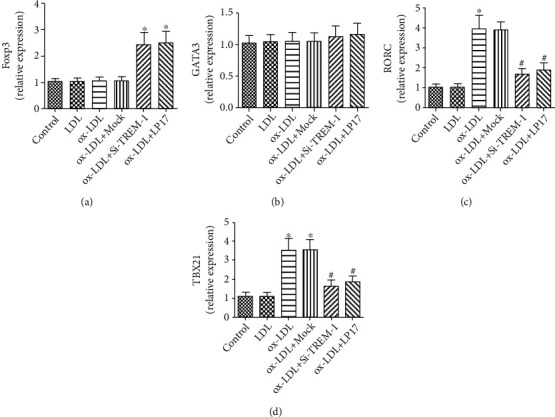
T-cell polarization-related transcription factors, TBX21, GATA3, RORC, and FOXP3 gene expressions, were evaluated by real-time PCR. ox-LDL-treated DCs strongly induced TBX21 and/or RORC expression in naïve T-cell populations. However, TREM-1 inhibition suppressed the expression of TBX21 and RORC while inducing the expression of FoxP3. Results represent the mean ± SD of seven experiments. ^∗^*p* < 0.05, compared with control cells; ^#^*p* < 0.05, compared with ox-LDL-treated cells.

**Figure 6:**
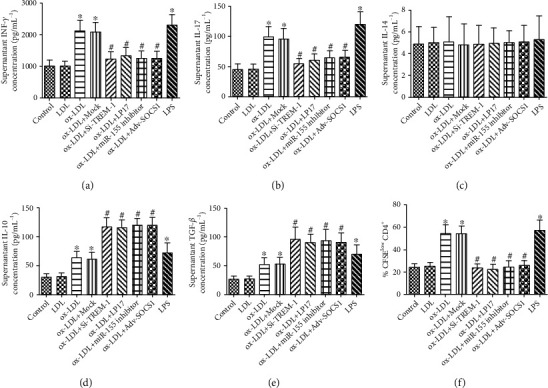
TREM-1 mediated the Th1/Th17-priming induced by ox-LDL-treated DCs. (a–e) IFN- *γ*, IL- 17, IL- 4, TGF-*β*, and IL- 10 content were determined by ELISA. (f) T [DC (siR-TREM-1 + ox-LDL)] cells suppressed primary T-cell proliferation. Naïve CD4^+^T cells were cultured with ox-LDL- or SiTREM-1 + ox-LDL-treated autologous DCs for 13 days. Naïve CD4^+^T cells labeled with 5 *μ*mol L^−1^ CFSES were stimulated with mature DCs (mDCs) alone or in the presence of T [DC], T [DC (ox-LDL)], T [DC (Si-TREM-1 + ox-LDL)], or T [DC (Mock + ox-LDL)] cells at a 1 : 1 ratio. T-cell proliferation was measured by CFSE dilution. Results represent the mean ± SD of seven experiments. ^∗^*p* < 0.05, compared with control cells; ^#^*p* < 0.05, compared with ox-LDL-treated cells.

Our results clearly demonstrated that compared with controls, blockade of TREM-1 significantly downregulated Th1/Th17 cell-priming cytokines and chemokines, including TBX2 (Th1), RORC (Th17), IFN-*γ*, IL-17, TNF-*α*, IL-1*β*, IL-6, MCP-1, CCL5, and CCL17, but upregulated Th2 cytokines (TGF-*β* and IL-10, GATA-3) (Th2) and expression Foxp3 (Treg) (Figures 5 and 6); however, the typical Th2 cytokines, IL-4 secretion, here, were observed in no significant differences (Figure 6(c)). On the whole, these data suggest that TREM-1 induces DCs maturation and stimulates their Th1/Th17-polarizing proinflammatory activity.

### 3.5. miR-155, SOCS1 Signaling Pathways Were Involved in the Effect of TREM-1

**Figure 7:**
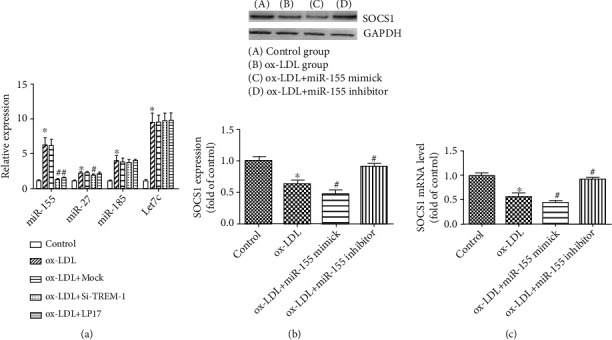
miRNA-155/SOCS1 signaling pathways were involved in the effect of TREM-1 and ox-LDL. (a) Expression of miRNAs in DCs was tested by RT-PCR. The value was normalized as a fold change to that of nontreated DC samples. (b-c) DCs were transfected with miRNA-155 inhibitor or mimic, respectively, and stimulated with ox-LDL. SOCS1 level in DCs were detected by Western blot (b) and RT-PCR (c). Results represent the mean ± SD of seven experiments. ^∗^*p* < 0.05, compared with control cells; ^#^*p* < 0.05, compared with ox-LDL-treated cells.

ox-LDL induced miR-155, miR-27, Let-7c, and miR-185 expressions. And miR-155 was the most prominent changed ones; however, after inhibition of TREM-1, the induction of miR-155 was abolished (Figure 7(a)).

SOCS1 was determined to mediate several proinflammatory cytokines. Here, we hypothesized that miR-155 might regulate its effects on the target of SOCS-1. The mRNA and protein expression of SOCS-1 were assessed by real-time PCR or western blot. As shown in Figures 7(b)–7(c), ox-LDL reduced the expression of SOCS-1 mRNA and protein expression, while cells pretreated with mimics or antagomir against miR-155 and next treated with ox-LDL decreased or increased the expression of SOCS-1 markedly. Furthermore, overexpression of SOCS-1 inhibited the production of TNF-*α*, IL-1*β*, IL-6, and MCP-1 in the DC cultures (Figure 4). These data show that miR-155 induced by TREM-1 regulates its effects through SOCS-1 downregulation and subsequently mediates proinflammatory cytokines production, including IL-1*β*, TNF-*α*, MCP-1, and IL-6.

### 3.6. TREM-1 Was Essential to the Ox-LDL-Induced Effect on DC Maturation, Cytokine Production, and T-Cell Proliferation Originated from Patients

**Figure 8:**
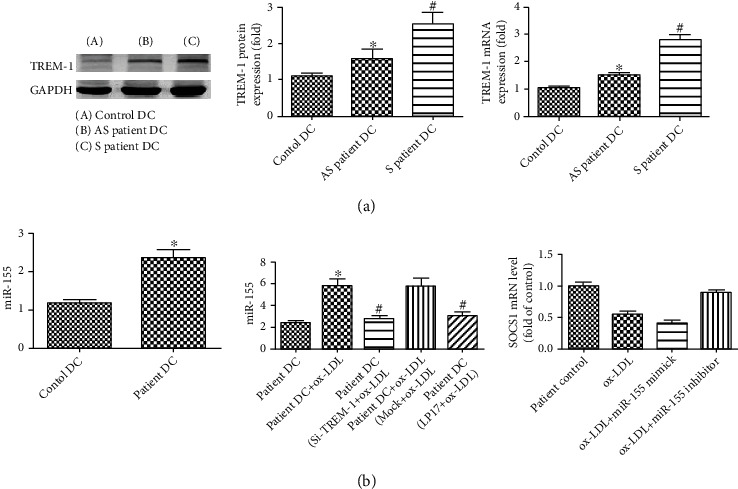
TREM-1 expression in DCs from symptomatic and asymptomatic patients with carotid stenosis and SOCS1 signaling pathways were involved in the effect of TREM-1 and ox-LDL on DCs from patients with carotid stenosis. (a) The mRNA and protein of TREM-1 expression in DCs from symptomatic were higher than from asymptomatic patients with carotid stenosis; (b) DCs were generated from peripheral blood monocytes of patients who underwent carotid endarterectomy. miR-155 and SOCS1 mRNA expression were determined by RT-PCR. ^∗^*p* < 0.05, compared with control cells; ^#^*p* < 0.05, compared with ox-LDL-treated cells.

**Figure 9:**
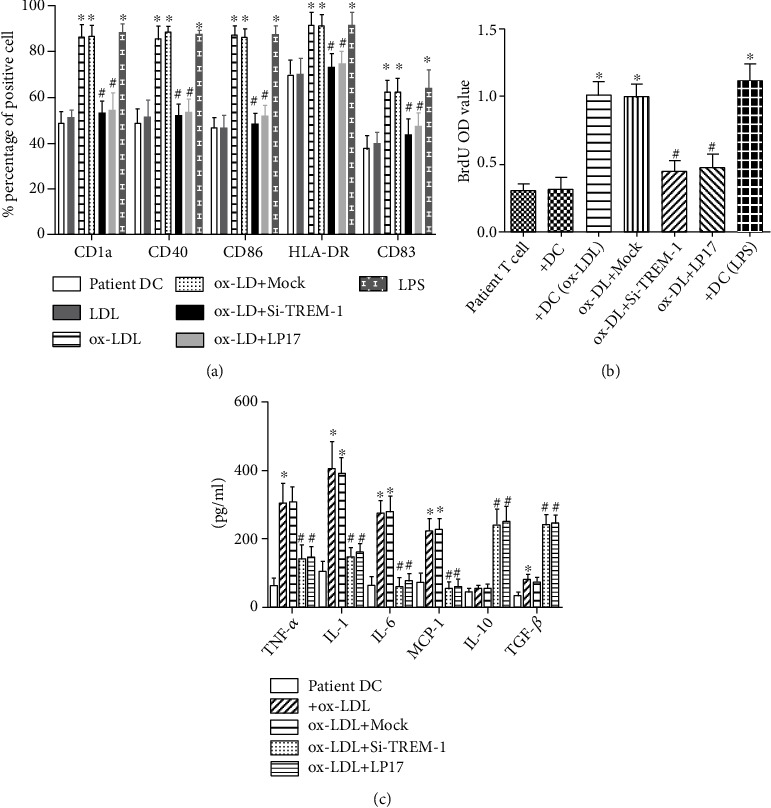
TREM-1 was involved in DC maturation, proinflammatory cytokine production, and T-cell proliferation in plaque patients induced by ox-LDL. DCs were generated from peripheral blood monocytes of patients who underwent carotid endarterectomy. The same protocol is used as that for DCs of normal donors. Briefly, DCs at day 6 were treated with 50 *μ*g mL^−^^1^ ox-LDL for 24 h. (a) The expressions of CD1a, CD40, CD86, CD83, and HLA‐DR on day 7 DCs were analyzed by flow cytometry. (b) T‐cell proliferation determined by BrdU incorporation assay. After the indicated treatment, DCs were washed and cocultured in triplicate for 5 days with autologous T cells. 10 *μ*mol L^−^^1^ BrdU was present in the last 16 h. Proliferative response was evaluated as OD value. (c) Cytokines production were tested by ELISA. Results represent the mean ± SD of seven-eight experiments. ^∗^*p* < 0.05, compared with control cells; ^#^*p* < 0.05, compared with ox-LDL-treated cells.

In relation to healthy donors, DCs from peripheral blood monocytes of patients who underwent carotid endarterectomy showed higher basal expression of TREM-1 (Figure 8(a)), CD1a, CD40, CD86, CD83, and HLA-DR (Figures 9(a)–9(d)) and higher basal amount of IL-6, IL-10, TNF-*α*, and TGF-*β* (Figure 9(f)). And by ox-LDL stimulation, they were further enhanced. However, pretreatment with LP17 or TREM-1 markedly decreased the expression of CD1a, CD40,CD86, CD83, and HLA-DR and inhibited TNF-*α*, IL-6, and MCP-1 secretion but increased IL-10 and TGF-*β* expression (Figures 9(a) and 9(c)).

Next, we explored the human plaque T-cell response to autologous ox-LDL-treated DCs. We found that ox-LDL-treated DCs, derived from patient peripheral blood monocytes, induced autologous plaque T-cell proliferation; however, the stimulated plaque T-cell proliferation was abolished by blocking TREM-1 with LP17 or TREM-1 siRNA treatment (Figure 9(b)).

## 4. Discussion

Here, we show that TREM-1 is upregulated by ox-LDL and orchestrates pivotal molecular events triggering DCs maturation and inflammatory factors production. Moreover, ox-LDL induces proinflammatory Th1/Th17 immune activation of DCs and T-cell activation, whereas inhibition of TREM-1 attenuates these effects. Next, we explore the mechanism, we found that TREM-1-induced miR-155 expression regulates its effects through SOCS-1 downregulation and subsequently mediates proinflammatory cytokines, including IL-1*β*, MCP-1, IL-6, and TNF-*α*. Thus, miR-155/SOCS-1 may play an important role in TREM-1-mediated inflammation and T-cell activation in ox-LDL-induced DCs.

Recent studies have proven that atherosclerosis is a chronic inflammatory disease [1–3]. Many research showed the functions of DCs in every step of atherosclerosis development, including lipid uptake, efferocytosis, antigen presentation, immune function, and chemokine secretion [35]. In the early formation of atherosclerotic lesions, the dendritic cells become foam cells by the uptake of lipoproteins and lipid-laden apoptotic cells, emigrating from the lesion into the draining lymph node to present antigens, as well as activate naïve T cells that are anti-inflammatory. In the advanced atherosclerotic lesions, the emigration of the dendritic cells from the plaque is defective; thus, they start to build up, which can lead to enhanced local inflammation [36]. In the present study, we indicated that ox-LDL sharply increases CD1a-, CD40-, CD86-, CD83-, or HL-DR- positive cells number, which mean the maturation of DC and inflammatory cytokines (IL-1*β*, IL-6, TNF-*α*, and MCP-1) secretion. These data indicated that ox-LDL induces DCs phenotype to make a harmful change and inflammatory response, which is accordance with previous studies.

TREM-1 is an immunoreceptor expressed mostly on neutrophils and monocytes/macrophages, and interestingly, recently, it is identified that acts on endothelial cells and regulates thrombin generation [37, 38]. Current evidence showed that TREM-1 appears to augment inflammatory responses in infectious diseases [39]. However, recently, TREM-1 has been verified to regulate noninfectious cardiovascular disease. In AMI models, TREM-1 is upregulated in the infarction zone, and inhibiting TREM-1 could effectively alleviate myocardial inflammation and improve heart function [21]. Moreover, TREM-1 was found to be expressed in atherosclerotic human lesions [40], and inhibition of TREM-1 alleviated atherosclerosis in a murine model [22]. Although TREM-1 was thought as a major upstream proatherogenic receptor, but its mechanism is not very clear; previous research showed that TREM-1 colocalized with DCs in atherosclerotic plaques in patients with carotid stenosis [31], which indicates that TREM-1 with DC may play key roles in the pathogenesis of atherosclerosis. In line with previous findings, we found that ox-LDL induced DCs maturation with increased expression of CD1a, CD40, CD86, CD83, and HLA-DR and inflammatory cytokines secretion, which could be abrogated by using TREM-1 siRNA or LP17 (an inhibitory peptide). Instead, TGF-b and IL-10 production were upregulated.

Another maturation process of DCs is the production of cytokines. Here, also as reported previously, T cells co-cultured with ox-LDL-treated DCs induced IFN-*γ*, IL-12, and IL-17 secretion, with polarization to Th1 and/or Th17 subsets [20]. Silencing TREM-1 abolished the DCs effects induced by ox-LDL and subsequent T-cell activation, with downregulation of transcription factor TBX21 and RORC expression, and induced T regulatory cells. T cells originated from healthy individuals or carotid atherosclerotic plaques showed similar results. Although the role of IL-17 and the corresponding Th17 cells in the pathogenesis of atherosclerosis are considered proatherogenic [41–43], the exact role of IL-17 and T17 cells in atherosclerosis is controversial. Tregs are now thought to regulate T-cell activation and suppress proinflammatory effects. This could inhibit foam cell formation and induce anti-inflammatory effects and subsequently ameliorate atherosclerosis, so increasing Tregs even were thought to be an interesting therapy for atherosclerosis and CVD [44]. These data here showed that ox-LDL induced T-cell activation mediated by DCs in vulnerable atherosclerotic plaques; however, TREM-1 silencing reversed these facts, suggesting that TREM-1 may play an important role in the development of atherosclerosis and plaque rupture by a novel-specific immune modulatory mechanism.

Over the past decade, microRNAs (miRNAs) have emerged as evolutionarily conserved, noncoding small RNAs that serve as important regulators in the progression and management of inflammation-related diseases, including atherosclerosis [23–25]. Although TREM-1 is known to exert inflammatory effects, whether TREM-1 also affects the expression of miRNAs in DCs and their role in inflammatory response of DCs induced by ox-LDL has remained elusive to date. MiR-155 has been shown to play important roles in immunity and inflammation, particularly in the inflammatory responses of DC, implying that it may also be involved in atherogenesis [26–29]. However, the function of TREM-1 on miR-155 expression in ox-LDL-stimulated inflammation remains unclear. In the current study, we observed that the expression of the inflammation-associated miR-155 was significantly increased in ox-LDL-stimulated DCS; in contrast, silencing of TREM-1 markedly decreased miR-155 expression (Figure 7(a)). Previous studies have shown that SOCS1 is one of the target genes of miR-155 [45–47]; our study also indicated that TREM-1 regulates miR-155 expression and the inflammatory response. Furthermore, mimics or antagomir against miR-155 downregulated or upregulated SOCS-1 expression markedly (Figures 7(b)–7(c)). These data show that TREM-1-induced miR-155 mediates its effects through SOCS-1 downregulation and subsequently mediates proinflammatory cytokines, including IL-1*β*, IL-6, MCP-1, and TNF-*α*.

In conclusion, we demonstrated that TREM-1 promoted dendritic cells maturation and T-cell activation and amplified the inflammation in DCs following ox-LDL treatment. Although previous studies showed that TREM-1 activation amplified monocyte/macrophage proinflammatory responses and induced foam cell formation to promote atherosclerosis [29], our studies gives evidence that TREM-1 could aggravate atherosclerosis not only by macrophage response, but at least partly by DC modulation. Additionally, we found that miR-155/SOCS1 pathway may play key roles in this process. This study provides molecular and biochemical evidence that inhibiting of TREM-1 may be a novel strategy and a promising target for the treatment of atherosclerosis.

## Data Availability

The data used to support the findings of this study are available from the corresponding author upon request.
